# Does the type of health insurance enrollment affect provider choice, utilization and health care expenditures?

**DOI:** 10.1186/s12913-020-05862-7

**Published:** 2020-11-03

**Authors:** Esso-Hanam Atake

**Affiliations:** Economics Department, University of Lomé, Lome, Togo

**Keywords:** Health insurance, Financial protection, Provider choice, Health service, Elderly, Togo

## Abstract

**Background:**

Two of the objectives of Universal Health Coverage are equity in access to health services and protection from financial risks. This paper seeks to examine whether the type of health insurance enrollment affects the utilization of health services, choice of provider and financial protection of households in Togo.

**Methods:**

Data were obtained from a cross-sectional, representative household survey involving 1180 insured households that had reported either illness in the household in the 4 weeks preceding the survey or hospitalization in the 12 months preceding the survey. A nested logit model was used to account for the utilization of health services and provider choice, and methods of assessing catastrophic health care expenditures were used to analyze the level of household financial protection.

**Results:**

Policyholders of private health insurance use private health care facilities more than policyholders of public health insurance. The main reasons for not using health centers among households with public insurance were out-of-pocket payments (49.19%), waiting time (36.80%), and distance to the nearest health center (36.76%). Furthermore, on average, households with public insurance spent a higher proportion of their total monthly nonfood expenditures on health care than those with private insurance. We find that the type of insurance, share of expenditures allocated to food, distance to the nearest health center, and waiting time significantly impact the choice of provider. Regardless of the type of health insurance, elderly individuals avoid using private health centers and referral hospitals due to the high cost.

**Conclusion:**

We found that a multiple health insurance system results in a multilevel health system that is not equitable for everyone. The capacity of the health insurance system to provide equitable health care services and protect its members from catastrophic health care expenditures should be at the core of health care reform. This study recommends raising awareness of the criteria for the reimbursement of medical procedures within the framework of public insurance and promoting specific health insurance mechanisms for elderly individuals. Careful attention should be paid to ensuring universal education and literacy as a means of improving access to and the use of health care.

## Background

Supply- and demand-side factors affect the utilization of health care services [1]. In some circumstances, the seeking of health care is mainly explained by the capacity to pay rather than the need for health care [1]. The reduction of social inequalities and the fight against poverty requires everyone to be able to access quality health services without incurring large financial burdens [2, 3]. To address this challenge, low-income countries are now accelerating reforms that promote health financing systems aimed at providing Universal Health Coverage (UHC).

UHC appears to be an appropriate solution to reduce unexpected health care costs, make intensive care accessible and strengthen the community’s sense of solidarity and willingness to provide equal access to health care for poor people [4]. Though this effect is predicted by economic theory, health insurance does not always lead to the expected financial protection [5]. Two main reasons explain the fact that health insurance may not achieve the desired objective. First, insurance benefits packages have a limited impact, especially regarding reimbursement levels, and therefore, while the introduction of health insurance could provide policyholders with better financial access to care, once they are involved in the system, the insurance fails to protect against accrued treatment costs [5, 6]. Second, through the introduction of a third-party payment mechanism, the service provider can encourage the patient to ask for increasingly more costly care that is different from what the patient would have chosen if he or she had had the same information as the provider [5, 7]. This situation raises the issue of the redistributive effects of health insurance schemes in Sub-Saharan Africa.

The Togolese health system is not spared from these issues in the implementation of UHC. The Togolese government embarked on a reform that resulted in the establishment of a National Health Insurance (NHI) scheme in 2011. The main reason for the implementation of NHI has been, as in other countries, the removal of financial barriers to adequate health care. Unfortunately, workers in the private and informal sectors are not covered by the NHI; at the same time, it has encouraged the development of private health insurance programs. This multiplication of health insurance programs raises questions of equity in the utilization of health care and financial protection services. The literature review reports that a multiple health insurance system generally results in a multilevel health system that is not equitable for everyone [8]. Private health insurance does not seem to be the solution against catastrophic health expenditures in Brazil [9]. The most successful health insurance programs are those that benefit the wealthiest groups [5]. Hidayat et al. [10] show that in Indonesia, the compulsory health insurance scheme for public-sector employees has a positive impact on access to public ambulatory care, while the compulsory insurance scheme for private-sector employees has a positive impact on access to both public and private ambulatory care. In China, the reimbursement scheme in place in each county and the average daily expenditure associated with hospitalization impact significantly hospital choice [11]. Moreover, it is reported that in rural China, out-of-pocket medical payments remain a burden for those households with the New Cooperative Medical Scheme [12]. Under the Iranian social security organization, utilization of health care services by insured persons not only relies on out-of-pocket, but it also depends on commands of general practitioners or specialist and/or geographical access [13]. For other scholar, distance appears to be one of the main influences on a patient’s choice [14].

These consistent issues that arise in sub-Saharan Africa, and more particularly in Togo, remain without solution. This study seeks to examine whether the utilization of health services, choice of provider and financial protection due to health insurance differ significantly from one group of policyholders to another, each group being characterized by their health insurance type. This study intends to help decision-makers in low-income countries, particularly those in sub-Saharan Africa, implement UHC. This paper seeks to provide important information for the implementation of strategies for improving the benefits of the Togolese NHI scheme. It addresses the two objectives of universal coverage: equity in access to health services and protection against financial risks.

A brief overview of the Togolese health insurance scheme is presented in the following section. The third section discusses the methods, while the fourth presents the results. Section five discusses the policy implications, and the last section concludes the paper.

### Mechanisms of health risk coverage in Togo

In Togo, there are many health insurance systems. The main ones are the national health insurance scheme, the private health insurance (commercial) system and the mutual health insurance system. On February 18, 2011, Act No. 2011–003 instituted the NHI scheme for public officials and their dependents via a participatory process initiated in 2009. The purpose of this scheme is to provide coverage for the risk of illness, accidents and nonoccupational diseases and for the maternity of public officials and their beneficiaries. The main objective of the NHI is to allow better access to quality care for policyholders of the health insurance scheme. This mandatory health insurance covers 80% of the costs of general and specialized consultations, pharmaceutical products, medical testing, medical imaging, nursing, and orthopedic appliances; 90% of the costs of hospital care; and 100% of the costs of healthcare services for pregnancy and childbirth. Health services in public health facilities are covered by the NHI; private health facilities, drug stores and eye care centers may apply for accreditation. In 2014, only 4.04% of the Togolese population was covered by the NHI [15, 16]. Thus, despite the efforts of the government, the majority of the population is not covered by National Health Insurance [15, 16].

As far as private health insurance is concerned, the main policyholders are private sector employees and their beneficiaries. The availability of coverage for the whole family and the level of payment offered for medical care depend on the capacity of the main insured to pay the related premiums. These types of insurance sometimes offer assistance services (such as advising), and their prices vary depending on the desired services. Their partners in the provision of health services are clinics, public health centers, pharmacies, laboratories and so on.

The mutual health insurance system is poorly developed in Togo. According to national health reports, the mutual health insurance system represents only 0.04% of health expenditures [17]. Togo is one of the countries with the fewest mutual health insurance programs among the West African states. Efforts are being made to promote the creation of mutual insurance companies within organized groups and businesses.

Furthermore, the government is making significant efforts to improve access to essential health care for the most vulnerable persons. These efforts include providing a subsidy to hospitals for the care of poor people; a state subsidy for cesarean births of up to 90% of the cost, which has been in effect since May 2010 as part of the efforts to reduce maternal and neonatal mortality; free antiretroviral drugs announced in November 2008; and free malaria care for all children under the age of 10 [18]. Moreover, all COVID-19 testing and treatment are free of charge in Togo.

Despite these efforts, utilization of health services has not improved in recent years. In 2017, the utilization rate for curative care was 39.20% [18]. An analysis of the supply of health services indicates major inequalities in the supply of and access to care. The levels of coverage for essential care differ substantially by environment and quintiles of economic well-being.

## Methods

### Modeling the utilization of health services and choice of provider

The analyses of the utilization of health services and choice of provider follow the behavioral framework of Gertler, Locay and Sanderson [19]. According to this model, utility depends on health and the consumption of goods other than medical care. This hypothesis assumes that when an illness or accident occurs, individuals must decide whether to seek medical care. The most important question that arises in the seeking of medical care concerns choosing a provider from a set of alternative providers. Each provider has a different impact on their health.

The utility function resulting from seeking medical care from provider *j* is defined by the following equation [19]:

1 $$ {U}_j=U\;\left({H}_j,{C}_j,{T}_j\right) $$

where *H*_*j*_ is expected health status after receiving treatment from provider *j*_,_ *C*_*j*_ is expenditures on consumption after paying provider *j*_,_ and *T*_*j*_ is the nonmonetary cost of access to provider *j*.

Similarly, Sahn, Younger and Genicot [20] suggest a model specification with five options: no care (self-care), care at a public hospital, care at a private hospital, care at a public clinic and care at a private clinic. In this paper, we use five options: *self-care/traditional healing, referral hospital (RH), district hospital (DH), peripheral health unit (PHU) and private health center (PHC)* [21]. Traditional healing or self-care refers to those who have recourse to traditional healers or pharmacies [8, 21].

To model health care utilization, several studies use multinomial or conditional logits. Such models assume that errors are independently and identically distributed (iid) and that the independence of irrelevant alternatives (IIA) condition is satisfied. However, a violation of such assumptions can result in inconsistent estimates [22]. The nested logit (NL) model, which reflects a choice framework such that individuals consider only the choices presenting the maximum utility for each decision, can be used in these cases [11, 14].

Following Heiss [23] and Brown and Theoharides [11], we use a two-level decision tree with *K* upper-level alternatives and *H* lower-level alternatives and define the utility function for patient/household *i* as:

*U*_*ih*_ = *R*_*ih*_ + *ε*_*ih*_, where

2 $$ {R}_{ih}={\alpha}_h+{\beta}_h{x}_{ih}+{\gamma}_h{y}_i $$

*R*_*ih*_, the deterministic portion of utility, is composed of the alternative specific variables, *x*_*ih*_, and the case-specific variable, *y*_*i*_. *ε*_*i*_ represents the random portion of utility and *h* ∈ *H*.

Then, the dissimilarity parameter is defined as ${\lambda}_k=\sqrt{1-{\rho}_k}$, where *ρ*_*k*_ represents the correlation of the alternatives within nest *k*, *k* ∈ *K*.

**Fig. 1:**
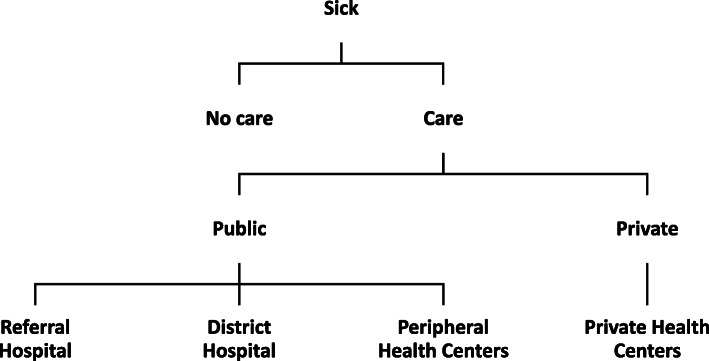
Estimated nesting structure

For the *K*^*th*^ level of the tree, the inclusive value parameter represents the utility that an individual *K* receives from making an alternative choice at this level of the tree (Fig. 1).

“The inclusive value parameters are the main differentiation between two types of models used in nested logit estimation, the nonnormalized nested logit model, and the random utility maximization (RUM) model” [11]. $$ {IV}_k=\ln \sum \limits_{h\in {D}_k}\exp \left({R}_{ihj}\right) $$ is the inclusive value in the nonnormalized nested logit model; *D*_*k*_ is the set of alternatives in choice level *k*, and *j* describes the number of choices available within that limb. In the RUM model, $$ {IV}_k=\ln \sum \limits_{h\in {D}_k}\exp \left(\raisebox{1ex}{${R}_{ihj}$}\!\left/ \!\raisebox{-1ex}{${\lambda}_k$}\right.\right) $$. In this case, the utilities are comparable between levels as they are scaled within each level by the dissimilarity parameter. In contrast, without scaling, utilities can only be compared for alternatives within the same level.

We therefore use the RUM model to specify a two-level nested logit model. We define *K* = {0, 1} as indices for whether the respondents seek healthcare, i.e., the limbs of the tree. Hospital choice (*H*) reflects whether sick individuals/households seek care in PHC, RH, DH or PHU or whether no care is sought. The top-level alternative (the choice of *K*) is denoted as *C*_1_ and the bottom-level alternative (the choice of *H*) as *C*_2_. The alternative chosen is the one with greatest utility. Errors in the nested logit model are assumed to follow the generalized distribution of extreme values to ensure correlation between the alternatives within the nest [24].

The conditional distribution of the random disturbances *ε*_*kh*_ is defined as:

3 $$ {F}_{H/K}\left(\varepsilon /k\right)=\left[-\left\{\sum \limits_{h\in {R}_k}\exp \left({\varepsilon}_{kh}/{\lambda}_k\right)\right\}\right] $$

Following Amemiya [22], the probability of choosing a particular provider *h*, given the choice of seeking care *k*, is defined as:

4 $$ \Pr \left({C}_2=j\left|{C}_1=k\right.\right)=\frac{\exp \left({x}_{kh}{\beta}_j/{\lambda}_k\right)}{\sum \limits_{m\in {R}_k}\exp \left({x}_{hm}{\beta}_m/{\lambda}_k\right)} $$

There are two general methods for normalization [25], one normalizing the scaling parameters to the lowest level and the other normalizing the scaling parameters to the highest level. The latter is consistent with utility theory [26]; hence, our results are based on that method. The estimations were performed using Stata.

The dependent variable is an indicator reflecting five categories of hospital choice (PHC, RH, DH, PHU, and none). The first-level alternative is estimated by seven case-specific explanatory variables according to Andersen’s behavioral model [27]. Andersen’s behavioral model identifies the predispositions, resources and characteristics of the needs of individuals/households as the main independent variables. Age, gender, education, health status, and household size are included in the predisposing characteristics. The variables used as proxies for resources are the type of employment and income. However, given the difficulty in measuring income, these variables are approximated by total household expenditures during the 4 weeks preceding the survey. Household expenditures devoted to food are included in the model as a proxy for household budget constraints [28].

### Measuring the incidence and intensity of catastrophic health expenditure

A catastrophic health expenditure (CHE) occurs when health-service payments by sick people or their households consume a very large portion of household available income [29]. These expenditures can push households into poverty or lead them into deeper poverty [30].

CHE has been described differently in the literature [29, 31–35]. The two key components underlying these different descriptions are out-of-pocket payments (OOPs) and household resources. Income alone cannot explain health care expenditures. In this study, the *capacity-to-*pay approach is used to compare CHEs across types of insurance. Two indicators are used: *the incidence of catastrophic payments* and *the catastrophic payment gap (intensity)*. According to O’Donnell [36], “the incidence of catastrophic payments can be estimated from the fraction of a sample with health care costs as a share of total (or non-food) expenditure exceeding the chosen threshold” [37]. The intensity of catastrophic payments represents the amount by which household OOP payments exceed the catastrophic threshold [37]. Food expenditure is used as a proxy for subsistence expenditure [12]. OOPs are defined as “direct payments made by individuals to health care providers at the time of service use. This excludes any prepayment for health services, for example, in the form of taxes or specific insurance premiums or contributions and, where possible, net of any reimbursements to the individual who made the payments” [38].

Let us suppose that no one should spend more than *z* nonfood expenditures on health care monthly. The capacity-to-pay is measured as follows:

5 $$ y=x-D(x) $$

where *x* is total monthly household expenditure and *D*(*x*) is food expenditure. Considering *T* as out-of-pocket payments (OOP) for health care, a threshold *z* can be determined for $\frac{T}{y}$. A variety of thresholds are used in the literature [29, 33]. For sensitivity issues, we use the following thresholds: 5, 10, 20, 30, and 40%.

We define an indicator *θ*_*i*_ (catastrophic overshoot) equivalent to $$ \frac{T_i}{y_i}-z $$ if $$ \raisebox{1ex}{${T}_i$}\!\left/ \!\raisebox{-1ex}{${y}_i$}\right.\succ z $$, and zero otherwise, and consider *E*_*i*_ = 1 if *θ*_*i*_ ≻ 0. Thus, the catastrophic payment is equal to:

6 $$ H=\frac{1}{N}\;\sum \limits_{i-1}^N{E}_1={\mu}_E $$

where *H* is the incidence of catastrophic payments, *N* is the sample size and *μ*_*E*_ is the mean of *E*_*i*_.

***The intensity of CHE*** is calculated using two measures, *overshoot and mean positive overshoot (MPO)* [39]. ***Overshoot*** shows the average degree to which OOP payments exceed the threshold *z*. Overshoot is calculated using the following formula:

7 $$ {O}_i={E}_i\left(\left(\frac{T_i}{y_i}\right)-z\right) $$

The average overshoot is:

8 $$ O=\frac{1}{N}\sum \limits_{i=1}^N{O}_i $$

***Mean positive overshoot (MPO)*** is defined as the payment in excess of the threshold averaged over all households exceeding that threshold. Thus, MPO is the overshoot divided by the proportion of households whose OOP is above the threshold.

9 $$ MPO=\frac{O}{H} $$

Regarding the measurement of catastrophic health expenditure, all expenditures were adjusted to be in terms of a single 4-week unit. For example, for expenditures on clothing, shoes, maintenance and repair, the reference period was the 3 months preceding the survey, while for expenditures including electricity, water, etc., the reference period was the month before the survey [40]. With regard to food expenditure, all information was collected for the 7 days preceding the survey [40]. As for health expenditure, the information collected includes expenses for medicines and vaccines, diagnostic costs and laboratory tests, consultation and treatment costs, hospital costs, costs of visits to traditional healers, transportation, and other health-related expenses in the 4 weeks preceding the survey.

### Study setting

This paper uses survey data from households covered with health insurance as part of the project to monitor the implementation of the National Health Insurance system in Togo by the Economics and Management Research and Training Center (CERFEG) of the University of Lomé (Togo) in collaboration with the African Population and Health Research Center (APHRC) and the International Development Research Center (IDRC). This study was conducted in the Lomé-Commune region (five districts). In health matters, there are six regions in Togo (Lomé-Commune, Maritime, Plateaux, Centrale, Kara and Savane), forty (40) districts and more than 882 peripheral health centers [30]. Although Lomé-Commune houses approximately one-quarter (24%) of the Togolese population, approximately three-quarters of the private health centers (76%) and health personnel (74%) and the highest concentration of households that have health insurance (40%) are found in this region [41]. While the five districts of Lomé-Commune are unique in some respects, the common characteristics include dwelling structures, access to piped water, health facilities, environmental sanitation, employment and income characteristics, education facilities, disease prevalence, and access to health insurance.

### Sample size

The WHO approach to two-sample situations (estimating the difference between two population proportions with specified absolute precision) was employed to determine the sample size [42].

With a 95% confidence interval, for a given difference in proportions, the sample size is given by:

9 $$ \left({p}_1-{p}_2\right)\pm 1.96\;\sqrt{\frac{p_1\left(1-{p}_1\right)}{n_1}+\frac{p_2\left(1-{p}_2\right)}{n_2}} $$

where *p*_1_ represents the incidence of CHE among households covered with public insurance and *p*_2_ the incidence of CHE among households with private insurance; *n*_1_ and *n*_2_ represent the size of each group.

If we suppose that the sample size in each group is the same, eq. 9 becomes the following:

10 $$ \left({p}_1-{p}_2\right)\pm 1.96\;\sqrt{\frac{p_1\left(1-{p}_1\right)+{p}_2\left(1-{p}_2\right)}{n}} $$

The values of *P*_1_and *P*_2_ were determined by referring to previous studies on the measurement of CHE. Barros et al. [9] show that among households with private insurance in Brazil, the CHE varied between 2 and 16%. In addition, in twelve countries in Latin America and the Caribbean, the percentage of households with CHEs varied between 1 and 25% [43]. From these references, we estimate that the incidence of households that are covered by public and private insurance and experience CHEs is 25 and 15%, respectively, in Togo.

With an absolute precision of 5%, the required sample size is 484 in each group, for a total of 968. To minimize sampling errors, the target sample size was increased. Thus, after calculating the sampling error, 590 insured households were required in each group, for a total of 1180 insured households. A representative sample was obtained by taking into account the density of the population by district as measured by the last general census of population and housing [44]. Thus, by district, 36, 490, 264, 84, and 306 households covered with health insurance were surveyed.

In addition, to avoid issues of under-power, some researchers recommend that power be computed retrospectively [45–47]. As limited as it might be, we calculated the post hoc power to confirm that the study is not severely underpowered. The estimated post hoc power for the two-sample means test is 0.9373 (Table 1). The test is significant, as the calculated post hoc power is higher than 0.5. Hence, we can conclude that the power calculation based on the difference in proportions is to some extent suitable to determine the appropriate sample size.

**Table 1:** Estimated post hoc power for a two-sample means test

*Study parameters*	
alpha	0.05
N	1180
N per group	590
delta	0.0301
m1	0.1197
m2	0.1498
sd1	0.1498
sd2	0.1468
*Estimated post-hoc power*	
Post-hoc power	0.9373

*Note:* m1 is mean of the share of OOP in private insured household’s monthly non-food expenditure; m2 mean of the share of OOP in public insured household’s monthly non-food expenditure; sd1 and sd2 respective standard errors;

### Sampling methods

The household survey was conducted from May 2 to 31, 2016. The questionnaire for this study was based on the 2002 World Health Survey and questionnaires from the 2013 Togo Demographic and Health Survey. Data were collected exclusively from insured households. Data were collected from households that had manifested at least one case of illness in the last 4 weeks before the survey or at least one case of hospitalization in the last twelve months before the survey. Moreover, the study collected data on residents (defined as members of a household with a minimum continuous stay of 3 months in the house) using a household-level survey conducted through an interviewer-administered questionnaire. In the absence of a population with health insurance enumeration listings, the households included in this study were selected using modified cluster sampling based on the segmentation of districts. Each district was divided into segments of approximately equal size, and the segments were all numbered. A random sample of the segments was taken, and from each of the selected segments, all households with health insurance were listed, and a random sample of households to be interviewed was taken from each selected segment [40].

### Data collection

The questionnaires include detailed expenditure and income questions for all insured household members. In addition, the questionnaire sought to capture information related to sociodemographic characteristics, household health status, access to health insurance, health care utilization behavior, water and sanitation, household livelihoods, coping strategies, and food and nonfood consumption. Expenditure and income data were collected in CFA Francs. The average exchange rate during the survey period, May 2 to 31, 2016, was 579 XOF per US dollar.

## Results

### Descriptive statistics of the sample

Table 2 indicates that households covered with public and private health insurance are similar along the dimensions of age, household size, and gender of the head of household. The summary of age shows that approximately 88.10 and 87.44% of heads of household who have public and private health insurance, respectively, were between 15 and 49 years of age. A high proportion of households with public insurance (44.07%) and private insurance (49.66%) had 2 to 3 members in the household. Among households with public insurance, approximately 60.78% of household heads have received higher education.

**Table 2:** Household characteristics and health-care-seeking behaviors

Variables	Public health insurance (***N*** = 590)	Private health insurance (*N* = 590)
*Household characteristics*		
*Household size (%)*		
1	107 (18.14)	92 (15.59)
2–3	260 (44.07)	293 (49.66)
4–5	138 (23.39)	108 (18.31)
6 -	85 (14.41)	97 (16.64)
*Age of household head*		
15–49	518 (88.10)	515 (87.44)
50–59	62 (10.54)	62 (10.54)
60 -	8 (1.36)	12 (2.04)
*Educational level of head of household (%)*		
No formal schooling	2 (0.34)	1 (0.17)
Primary	19 (3.23)	86 (14.97)
Secondary school (Junior and senior)	209 (35.48)	496 (84.36)
High level (university)	358 (60.78)	3 (0.51)
*Gender of household head (%)*		
Female	167 (28.13)	187 (32.02)
Male	419 (71.87)	397 (67.98)
*Household’s expenditures*		
Mean household total expenditure (FCFA)	319, 825	247, 127
Mean OOP health expenditure (medical & nonmedical) (FCFA)^a^	31, 657.05	24, 042.4
Mean household food expenditure (FCFA)	110, 748.4	85, 078.47
*Health care seeking behavior*		
*Use oh health care (illness) (%)*		
Yes	390 (66.10)	378 (64.07)
No	200 (33.90)	212 (35.93)
*Reasons for not seeking health care from a medical facility (%)*		
Distance	68 (36.76)	98 (48.51)
Waiting time	68 (36.80)	27 (13.37)
Low quality healthcare	48 (25.95)	47 (23.27)
Out-of-pocket payments	91 (49.19)	86 (42.57)

^a^*The FCFA is the name of the currency used in part of West African countries including Togo*

Households with public insurance spent a monthly average of 319,825 FCFA (552.37 USD) compared to 247,127 F CFA (426.82 USD) for those with private insurance. Monthly out-of-pocket payments were estimated at 31,657 F CFA (54.67 USD) and 24,042 F CFA (41.52 USD), respectively, for households with public and private insurance.

### Health-seeking behavior and health care utilization

Table 2 reveals that approximately 66.10 and 64.07% of households with public and private insurance, respectively, resorted to health centers in the event of illness. The main reasons for not resorting to health centers among households with public insurance were out-of-pocket payments (49.19%), waiting time (36.80%), and distance to access a health center (36.76%). Among those with private insurance, the main reasons mentioned were distance (48.51%), out-of-pocket payments (42.57%), and the quality of health services (23.27%).

### Choice of health care provider

Table 3 shows that among those who resort to health care facilities, the choice of provider varies according to the type of insurance. Households with private insurance use more private health centers, while those with public insurance use more public health centers. Regardless of the type of insurance, the quality of the service (availability of drugs, availability of diagnostic and laboratory tests, health personnel, etc.) and distance are the main factors that determine the choice of the provider. Notably, the two-sample test of proportions shows that the proportion of private-insurance policyholders and public-insurance policyholders are significantly different from each other for most variables. Additionally, we find evidence of an association between the type of health insurance and the main reason determining provider choice (chi-2 test; Table 3).

**Table 3:** Distribution of households by provider and the determinants of choice

Variables	Public health insurance (*N* = 590)		Private health insurance (*N* = 590)		Difference between the
	Proportion (%)	chi-2 test	Proportion (%)	chi-2 test	two proportions
*Public health centers (referral Hospitals)*	***N = 89 (22.82)***		***N*** **= 76 (20.11)**		
Distance	13.79	28.32***	23.68	26.63***	−0.45***
Health staff offer good advice	6.90	13.62***	6.58	8.05**	−0.51***
Waiting time	1.15	21.26***	3.95	14.30***	− 0.49***
High-quality healthcare	40.23	21.08***	36.84	11.97***	−0.25***
Affordable healthcare	14.94	32.86***	14.47	8.01**	−0.33***
*Public health centers (districts hospitals)*	***N = 130 (33.33)***		***N*** **= 54 (14.29)**		
Distance	48.46	8.19**	67.31	21.79***	−0.30***
Health staff offer good advice	0.00	23.27***	1.89	6.96*	−0.63***
Waiting time	4.62	17.15***	5.66	1.89	−0.45***
High-quality healthcare	15.38	17.22***	15.09	15.58***	−0.38***
Affordable healthcare	1.54	21.17***	1.89	16.81***	−0.54***
*Public health centers (Peripheral health centers)*	***N = 72 (18.46)***		***N*** **= 49 (12.96)**		
Distance	70.83	35.13***	61.22	22.44***	−0.34***
Health staff offer good advice	4.17	3.98	10.20	7.66*	−0.55***
Waiting time	15.28	8.88**	8.16	3.26	−0.40***
High-quality healthcare	16.67	28.95***	20.41	3.96	−0.40***
Affordable healthcare	2.82	3.77	8.16	1.75	−0.51***
*Private health centers (CSP)*	***N*** **= 99 (25.38)**		***N*** **= 199 (52.65)**		
Distance	31.63	10.46**	30.26	20.02***	−0.32***
Health staff offer good advice	7.14	2.49	10.31	24.62***	−0.32***
Waiting time	22.45	29.02***	13.92	18.74***	−0.12*
High-quality healthcare	28.57	0.98	20.10	8.20**	−0.24***
Affordable healthcare	6.12	6.28*	8.25	23.12***	−0.35***

*** Significant at 1%; ** significant at 5%; * significant at 10%

### Determinants of resorting to health care and choice of provider

The results for the two-level nested logit model of health care choice are shown in Tables 4 and 5. The results are separated into two nests based on the decision tree, with the top nest reflecting the decision to seek health care (Table 4) and the bottom nest reflecting the choice of hospital (Table 5). First, the results of Hausman’s specification test [48] for the IIA (independence of irrelevant alternatives) are good. We cannot reject the IIA at the commonly used 1% significance level. This test suggests that, because the errors are i.i.d., they cannot contain any alternative-specific unobserved information, and therefore, adding a new alternative cannot affect the relationship between a pair of existing alternatives. Moreover, we examined the variance inflation factors (VIFs). The highest VIF value is 8.74, and the average VIF is approximately 1.98, suggesting low multicollinearity.

**Table 4:** Determinants of health care utilization, estimated with nested logit model (upper-level alternatives)

Variables	
*Household size*	
2–3	0.90^a^ (0.47 1.33)
4–5	1.29^a^ (0.74 1.85)
6 -	1.01^a^ (0.44 1.59)
*Educational level of head of household*	
Primary	2.90^b^ (0.59 5.21)
Secondary school	2.41^b^ (0.41 4.42)
High level (university)	2.62^b^ (0.61 4.64)
*Gender of household head*	
Male	0.33^c^ (−0.03 0.69)
*Age oh household head*	
50–60	−0.50^a^
60 –	−0.02
*Employment status of household head*	
Public sector	0.57 (−0.72 1.30)
Private sector	−0.12 (−1.10 0.87)
Parastatal sector	−0.33 (−1.37 0.71)
*Having a family member with a chronic illness*	
Yes	0.85^a^ (0.49 1.21)
*Household’s total expenditure*	6.40e-07^c^ (−1.14e-07 1.39e-06)
*Number of Observation*	1180
LR test for IIA (tau = 1): chi2(1) = 9.07 Prob > chi2 = 0.0026	

*Note:* ^a, b, c^ *significant at 1, 5, and 10%*

*Coefficients are reported as odds-ratios and confidence interval are shown in parentheses*

**Table 5:** Determinants of hospital choice, estimated with nested logit model (bottom-level alternative)

Variables	PHC^**a**^	RH^**b**^	DH^**c**^	PHU^**d**^
*Type of insurance*				
Private insurance	0.34** (0.02 0.81)	0.48** (0.17 1.42)	1.63 (0.21 3.04)	1.01 (0.11 1.91)
Share of expenditures allocated to food	−1.95** (−3.51–0.40)	−1.72* (−3.62 0.16)	−4.28*** (−6.62–1.94)	−2.02** (− 3.76–2.29)
*Health care quality*				
Good health care quality	−0.25 (− 0.79 0.27)	−0.33 (− 0.97 0.30)	0.67 (− 0.33 1.66)	−1.25 (− 0.90 0.40)
*Waiting time*				
Long waiting time	−1.45*** (− 1.97–0.92)	−0.26 (− 0.89 0.40)	−1.64*** (− 2.24–1.03)	−1.26*** (− 1.85–0.67)
*Distance to the nearest health center*				
*Health facilities’ locations close to population*	0.32 (− 0.18 0.83)	−1.06 (− 3.02 0.89)	1.38** (0.07 2.69)	2.02* (− 0.03 4.07)
*Age of household head*	−0.07* (− 0.14 1.96)	−0.17* (− 0.23 0.18)	−0.43 (− 0.67–0.02)	−0.04 (− 0.27 0.21)
Number of observation	768			
LR test for IIA (tau = 1): chi2(1) = 9.07 Prob > chi2 = 0.0026				

*** Significant at 1%; ** significant at 5%; * significant at 10%

^*a*^ *Private health centers* ^*b*^ *Referral hospitals* ^*c*^ *District hospitals* ^*d*^ *Peripheral health units*

*Coefficients are reported as odds-ratios and confidence interval are shown in parentheses*

We find that the likelihood of patients seeking health care increases as the head-of-household’s education level and the household size increase (Table 4). Next, having a household member with chronic disease positively impacts the probability that care is sought at the 99% confidence level. High household total expenditure also increases the odds that an individual seeks health care. However, we find that households with older people are less likely to seek care. We do not find a significant effect from the employment status of the household head.

The results in the bottom nest are relevant for policy as they explain where insured households seek health care conditional on seeking treatment (Table 5). We find that households with private insurance are more likely to use PHCs and RHs than those who are enrolled in public insurance. Regardless of the health center chosen, the share of expenditures allocated to food has a negative and significant relationship with the choice of provider. The probability of using RHs decreases as the age of the head of household increases. Although the coefficients associated with other types of health centers are not statistically significant, their signs suggest that an increase in age leads to a decrease in attendance at health centers. Furthermore, health facility locations close to the population have a positive effect on DH and PHU attendance. Finally, the likelihood of using PHCs, DHs and PHUs decreases with waiting time.

### Incidence and intensity of catastrophic health expenditure (CHE)

Table 6 indicates that the proportion of households facing CHEs does not vary significantly by type of insurance. Among households that have public insurance, the proportion of households facing CHEs varies from 62.36 to 4.16% when the threshold varies from 5 to 40%. For private policyholders, the proportion of households facing CHEs varies from 61.15 to 3.84% as the threshold varies from 5 to 40%. If we increase the threshold from 5 to 40%, then the mean overshoot (extent by which households exceed a given threshold) drops from 8.13% of expenditure to 1.04% among households that have public insurance and drops from 8.70% of expenditure to 0.96% among households that have private insurance. The mean positive overshoot (MPO) reveals that among publicly insured households, those spending more than 25% of nonfood expenditure, on average, spent 42.47% (25% + 17.47%) on health care, whereas those spending more than 40% of their nonfood expenditure, on average, spent 64.94% (40% + 24.94%) on health care.

**Table 6:** Proportion of households experiencing catastrophic health expenditure (*N* = 1180)

Threshold, z (%)	5	10	15	25	30	40
	**Policyholders of public health insurance**					
% Head count	62.36 (0.485)	40.70 (0.492)	24.95 (0.433)	11.82 (0.323)	7.66 (0.267)	4.16 (0.20)
% Overshoot	8.13 (0.139)	5.56 (0.127)	3.94 (0.114)	2.06 (0.09)	1.61 (0.08)	1.04 (0.06)
% Mean Positive Overshoot	13.03 (0.157)	13.67 (0.169)	15.79 (0.183)	17.47 (0.209)	21.04 (0.216)	24.94 (0.208)
	**Policyholders of private health insurance**					
% Head count	61.15 (0.488)	42.45 (0.495)	29.30 (0.459)	15.11 (0.358)	10.31 (0.304)	3.84 (0.192)
% Overshoot	8.70 (0.141)	6.14 (0.127)	4.34 (0.114)	2.21 (0.09)	1.61 (0.079)	0.96 (0.063)
% Mean Positive Overshoot	14.24 (0.156)	14.46 (0.162)	14.49 (0.169)	14.64 (0.187)	15.60 (0.201)	25.14 (0.216)

(): standard deviation

## Discussion

This study examines the impact of the type of health insurance enrollment on the choice of provider, health service utilization and health care expenditures.

The following determinants all significantly impact health care utilization: the head-of-household’s education level, household size, presence of a household member with chronic disease, household total expenditures, and the head-of-household’s age.

It is noteworthy that the increase in the size of the household significantly increases the likelihood of seeking health care. The households’ welfare and standard of living could be influenced by the size of the household. The size of a household could imply a significant economic burden on families. Therefore, for households with health insurance, utilization of health care could reduce the economic burden, as a significant part of the health expenditures would be covered by the insurer. This result corroborates those of Muriithi [49], who found that in Kenya, household size was positively correlated with health care utilization in formal health centers. Similarly, in Benin, having a large family increases the probability of resorting to public and private health centers rather than using self-medication [50].

As expected, the level of education of the household head positively affects the demand for health care. The education level of the household head proxies for knowledge of health. Heads of household with high levels of education have better knowledge of the usefulness of health care services and have a greater ability to communicate effectively with health care providers [51]. Education also plays a significant role in raising awareness of health issues, influencing beliefs about disease causation and means of cure, as well as impacting the use of modern health care facilities. Moreover, the level of education of the household head influences the expected health status of household members and therefore the use of formal health centers. Our results corroborate those from studies of households in sub-Saharan Africa, such as those of Eme Ichoku and Leibbrandt in Nigeria [52], Muriithi in Kenya [49], and Cissé et al. [53] in the Ivory Coast. These results are, however, in contradiction to those of Gertler and Van der Gaag [54], who showed that the level of education of the head of household had no statistically significant relation with the seeking of health care. Our results suggest that careful attention should therefore be paid to ensuring universal education and literacy as a means of improving access to and the use of health care.

Similarly, the presence of a household member with a chronic illness positively affects health care utilization. Chronic diseases are associated with poor functional status [55], poor quality of life [56–58], increased psychological distress [59] and mortality [60]. The health of people with chronic diseases is disproportionately complex and difficult for them to manage, and thus, such people make intensive use of care [57]. Hence, chronic diseases are generally associated with higher levels of health care utilization. Chronic diseases and the consequential burden on financial and human resources provide incentives for insured households to use formal health care.

In addition, our results show that household total expenditures positively impact health care utilization. In this study, we used household consumption expenditures, which are widely considered to be a more reliable measure of household wealth than self-reported income [61]. Consumption expenditures also capture the economic capacity of many households in developing countries. Our results show that the most advantaged with regard to household expenditures use more health care services than the least advantaged. A growing number of studies worldwide indicate that low household wealth is associated with poor health status and lower use of health care [62]. High-income households are more likely to participate in regular health check-ups and to receive health-related educational opportunities [62]. Therefore, a high standard of living increases the likelihood of seeking health care, as well as the magnitude of health expenditures.

As far as the choice of health care provider (choice of hospital) is concerned, important policy implications are derived from the results of this study. We find that the type of insurance, share of expenditures allocated to food, distance to the nearest health center, age of household head, and waiting time all significantly impact the choice of provider.

The likelihood of resorting to PHCs is higher among households enrolled in private insurance. The main reason for this difference in resorting to health services is explained by the fact that private insurance programs mainly offer health services for their policyholders in private clinics, pharmacies and laboratories and pay fees to medical doctors. On the other hand, public insurance provides health care to public sector employees mainly in public health care facilities. However, households enrolled in public insurance have the option of using private health centers, provided that they have been previously approved by National Health Insurance. A total of 196 PHCs, i.e., less than 25% of PHCs and pharmacies were approved by the NHI in 2016 [28]. Because private insurance offers an increasingly wide range of services based on capacity-to-pay, private insurance policyholders seek more PHC services than public health center services. Public health centers often struggle with taking care of long-term diseases, providing surgeries, conducting medical evacuations, following up with care abroad, etc.

Moreover, some health practitioners in certain PHCs approved by the NHI are reluctant to accept households covered with public insurance [15]. Their reluctance is justified by the reimbursement processes of hospitals. Delays in reimbursement for hospitals lead to the refusal by PHCs of medical care vouchers for households covered by public insurance. Our results suggest that appropriate measures need to be taken to ensure that hospitals are reimbursed in a timely manner. Furthermore, with regard to reimbursement criteria, there is also a conflict between the PHCs and the NHI. Medical procedures and drugs are provided and reimbursed according to 3 criteria: kind of disease, level of effectiveness and the profit/risk ratio. Unfortunately, different stakeholders have different understandings of these criteria. A policy implication of this study is that decision-makers should raise awareness regarding the rules of medical care and the criteria for the reimbursement of medical procedures and medicines.

Another important result concerns elderly patients. The results show that the likelihood of using RHs and PHCs decreases as the head-of-household’s age increases. Regardless of the type of insurance, households headed by elderly people make increasingly less use of hospitals, particularly RHs, which are theoretically supposed to treat chronic diseases, severe cases, and emergencies and to provide intensive care, continuity of care, and surgeries, which are particularly common among elderly patients. In a situation where among public insured households those spending more than 30% of nonfood expenditure, on average spent 51.04% on health care, elderly patients (the majority of whom are pensioners) would not be encouraged to use modern health care centers, as shown in Table 5. Our results corroborate those of Biswas et al. [63], who found that elderly people in Bangladesh did not use qualified health workers because of the high costs. These results suggest that decision-makers propose a specific health insurance policy for elderly patients. To encourage the utilization of hospitals and limit the impoverishment of elderly individuals, free or highly subsidized hospital service policies should be planned.

In addition, the likelihood of recourse to all types of health care centers decreases as the share of expenditures allocated to food increases. Clearly, as more of the budget is allocated to food, less money is available to spend in recourse to formal health centers. Obviously, in the context of resource scarcity, the more households allocate of their budget for food expenditure, the less financial means they will have to seek medical care. The budget allocated to food expenditure represents a major constraint in terms of the utilization of formal health centers. Our results corroborate those of Brown et al. [64] and Makinen et al. [28].

Furthermore, these results present a positive and significant relationship between distance and seeking care in DHs and PHUs. These results indicate that the distance to health centers is a major obstacle to seeking medical care, particularly in peri-urban and rural areas, where DHs and PHUs are located. Peri-urban residents generally suffer from a shortage of health care providers, prolonged travel, low socioeconomic status and lack of social support. Bringing health centers closer to the population could increase the utilization of health care and improve the health status of the population. This finding corroborates the results of Musoke et al. [65] and of Prosser [66], who showed that distance to health centers was one of the major challenges in Uganda and Kenya, respectively. On the other hand, waiting time also negatively impacts the recourse to health care in PHCs, DHs, and PHUs. As reported in the descriptive statistics, the waiting time for an appointment is ranked among the most important factors for patients when choosing a hospital. It seems reasonable that some patients would be happy to go to a more distant hospital if it would reduce their waiting time [14]. It is interesting to note that a study comparing the trade-off between waiting time and traveling distance showed that patients are willing to travel to hospitals that are far away if the waiting times at those hospitals are decreased [14, 67]. Our results confirm those from papers that demonstrate that both distance from the hospital and waiting times have a strong impact on how patients choose their hospital [14].

This study has certain limitations. It focuses on insured households in a single region. In addition, the lack of information on the type of private insurance companies available and other factors specific to different providers, such as the quality of care, are also limitations. The expenditure data used to measure the various indicators have been self-reported and have not been verified from other sources. The existence of measurement error in the evaluation of out-of-pocket payments and catastrophic health expenditure should not be ignored. Finally, it is important to mention that our results do not allow us to determine whether the difference in the choice of medical provider is the result of a difference in the quality of care among providers.

## Conclusion

In this paper, we examined whether the type of health insurance enrollment affects the utilization of health services, the choice of provider and the financial protection of households in Togo. A nested logit model and catastrophic payment methods were used to achieve this objective. We find that the head-of-household’s education level, household size, presence of a household member with a chronic disease, household total expenditure, and presence of older adults in the household all affect the decision to seek health care. These significant results also reveal that households with private insurance use more medical services in private health centers than those with public insurance. These results also indicate that elderly patients avoid using PHCs and RHs because of their high costs. Our results suggest that careful attention should be paid to ensuring universal education and literacy as a means of improving access to and the use of health care. Decision-makers should raise awareness regarding the rules of medical care and the criteria for the reimbursement of medical procedures and medicines. To encourage the utilization of hospitals and limit the impoverishment of elderly individuals, free or highly subsidized hospital service policies should be planned. Finally, bringing health centers closer to the population could increase the utilization of health care and improve the health status of the population.

## Data Availability

The data supporting the conclusions of this article are available at the Directorate of Research data repository of the University of Lomé and can be obtained with a written permission.

## Abbreviations

CHE: Catastrophic health expenditure
DH: District Hospital
MPO: Mean Positive Overshoot
NHI: National Health Insurance
PHC: Private Health Center
PHU: Peripheral Health Unit
RH: Referral Hospital
UHC: Universal Health Coverage
VIF: Variance Inflation Factors
